# Proposal of a Method to Determine the Correlation between Total Suspended Solids and Dissolved Organic Matter in Water Bodies from Spectral Imaging and Artificial Neural Networks

**DOI:** 10.3390/s18010159

**Published:** 2018-01-09

**Authors:** Maurício R. Veronez, Lucas S. Kupssinskü, Tainá T. Guimarães, Emilie C. Koste, Juarez M. da Silva, Laís V. de Souza, William F. M. Oliverio, Rogélio S. Jardim, Ismael É. Koch, Jonas G. de Souza, Luiz Gonzaga, Frederico F. Mauad, Leonardo C. Inocencio, Fabiane Bordin

**Affiliations:** 1 Advanced Visualization & Geoinformatics Lab—VizLab, Unisinos University, São Leopoldo 93022-750, Brazil; emiliek@edu.unisinos.br (E.C.K.); lgonzaga@unisinos.br (L.G.J.); lcinocencio@unisinos.br (L.C.I.); fabianebor@unisinos.br (F.B.); 2 Graduate Programme in Geology, Unisinos University, São Leopoldo 93022-750, Brazil; laisvs@edu.unisinos.br; 3 Graduate Programme in Applied Computing, Unisinos University, São Leopoldo 93022-750, Brazil; lkupssinsku@edu.unisinos.br (L.S.K.); juarezmachado@edu.unisinos.br (J.M.d.S.); williamoliverio@edu.unisinos.br (W.F.M.O.); rogeliosj@edu.unisinos.br (R.S.J.); iekoch@edu.unisinos.br (I.É.K.); jonassouza@edu.unisinos.br (J.G.d.S.); 4 Graduate Programme in Environmental Engineering Sciences, São Carlos Engineering School, University of São Paulo, São Carlos 13566-590, Brazil; tainathomg@usp.br (T.T.G.); mauadffm@sc.usp.br (F.F.M.)

**Keywords:** spectral imaging, unmanned aerial vehicles, correlation, water quality monitoring, artificial neural networks

## Abstract

Water quality monitoring through remote sensing with UAVs is best conducted using multispectral sensors; however, these sensors are expensive. We aimed to predict multispectral bands from a low-cost sensor (R, G, B bands) using artificial neural networks (ANN). We studied a lake located on the campus of Unisinos University, Brazil, using a low-cost sensor mounted on a UAV. Simultaneously, we collected water samples during the UAV flight to determine total suspended solids (TSS) and dissolved organic matter (DOM). We correlated the three bands predicted with TSS and DOM. The results show that the ANN validation process predicted the three bands of the multispectral sensor using the three bands of the low-cost sensor with a low average error of 19%. The correlations with TSS and DOM resulted in R^2^ values of greater than 0.60, consistent with literature values.

## 1. Introduction

Water quality monitoring involves data and water sample collection in the field and subsequent laboratory analysis. The effectiveness of the monitoring efforts depends on several factors, such as the frequency of sampling and the spatial distribution of parameters considered in the analysis [1,2,3]. Conventional water collection and analysis techniques are often costly and time-consuming, and may render water quality monitoring projects unfeasible. This is exacerbated by the locations of many water bodies, which may be in places that are difficult or dangerous to access, making on-site monitoring impossible. Therefore, there is a need to develop reliable and cost-effective spatial techniques for monitoring water quality that can be easily deployed [4].

The assessment of water quality based on remote sensing techniques is not new [5,6]. According to [7], several types of satellite-borne sensors have been used in the last three decades for monitoring aquatic environments. However most of these studies are based on assessing the quality of marine and coastal waters [5,6,8,9].

Challenges to advancing sustainable water quality management practices include the requirement for rapid and accurate monitoring and assessment of water quality in rivers, reservoirs, lakes, and estuaries, as well as knowledge of their spatiotemporal distribution [10,11,12]. Using appropriate technologies to effectively monitor these natural resources is, therefore, necessary. Remote sensing could be a complementary or alternative tool for monitoring water resources.

Remote sensing methods measure the changes in the spectral behavior of optically active components (OACs) in a water body, based on the variations in their concentration. OACs can interact with electromagnetic radiation (EMR), influencing the spectral behavior of water with their own spectral signatures, and altering its transparency. Such changes can be identified by sensor systems [12]. These substances can be identified through remote sensing techniques because their presence in a water body results in different EMR absorption and backscattering patterns, which are characteristic of each component. Suspended sediments and dissolved organic material are among the main parameters that influence the absorption and scattering of EMR in a water body [13].

Although satellite remote sensing technologies are effective in estimating the quality of water in large bodies, they still have the following limitations: (a) atmospheric interference in the images, affecting data interpretation and leading to a loss of confidence in the estimated water quality parameters; (b) low spatial resolution, making the detection of water pollution in small areas difficult; and (c) inefficient image review cycles that do not meet the need for rapid monitoring of these environments [11].

Sensors mounted on unmanned aerial vehicles (UAVs) have received increasing attention because they can provide high spatial resolution images, allow for the constant monitoring of the environment [14], and access the places that would otherwise be difficult or dangerous to visit [15]. Spectral sensors can be mounted on UAVs to provide images in different channels of the spectrum, which can be processed by various methods and algorithms, generating point clouds and surface models for various applications [16].

UAVs have been used to monitor the concentration of chlorophyll-a [17,18,19] and aquatic vegetation [20,21], spatialize suspended sediments [1], conduct environmental inspections [22], study flood events [23], conduct morphological and hydraulic characterizations [24], and monitor water pollution [25]. However, few studies have focused on determining the spectral behavior of water bodies using UAVs to estimate the concentration of multiple OACs. This is primarily due to the high costs of multi/hyperspectral sensors, which are often valued at USD 10,000 and USD 40,000.

Low-cost compact sensors are currently available on the market (~USD 600), with a modified filter sensitive to near-infrared radiation. This filter does not allow the passage of red-wavelength radiation (approximately between 600 and 700 nm), but it captures all other frequencies, including infrared, generating a charge-coupled device (CCD) with red channels that respond to the infrared band. These sensors have commonly been used in precision agriculture.

Thus, (a) to monitor water quality, we need multi/hyperspectral sensors that are sensitive to the electromagnetic spectrum between the visible and near infrared (400 to 800 nm) regions; (b) multi/hyperspectral sensors that can be used in UAVs are very expensive; and (c) low-cost sensors are not sensitive to the bands of the electromagnetic spectrum that are important for water quality monitoring.

These issues motivated us to propose an alternative method, based on an expert system for monitoring water quality using UAVs. We established the following hypothesis: it is possible to predict bands in the sensitive spectrum to monitor water quality using a low-cost sensor sensitive to the blue (B), green (G), and red (R) spectral bands. The proposed expert system is an artificial neural network (ANN).

## 2. Materials and Methods

**Figure 1:**
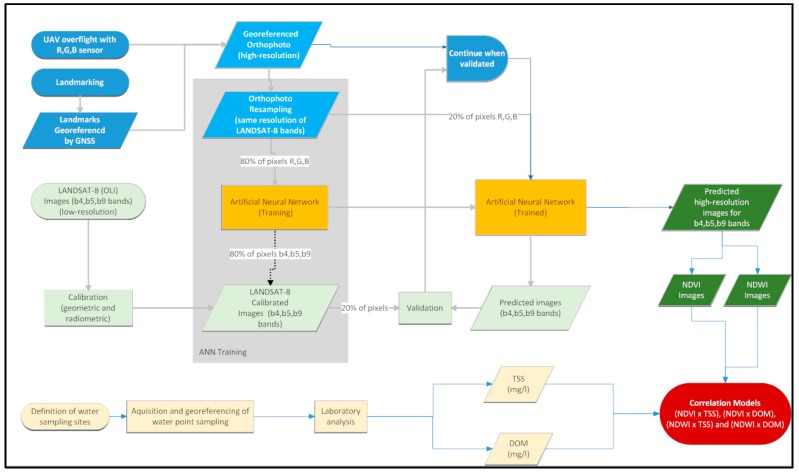
Flowchart of the proposed method.

The method that we are proposing can be structured according to the following steps: overflight with the UAV and processing of the acquired images; LANDSAT 8 (OLI) image processing; collection of water samples and laboratory analysis; training and validation of the ANN; generation of *NDVI* and *NDWI* images by the ANN; and establishment of mathematical models to correlate the *NDVI* and *NDWI* indices with TSS and DOM. The flowchart of the proposed method is depicted in Figure 1 and detailed in the following items.

### 2.1. Field Site

**Figure 2:**
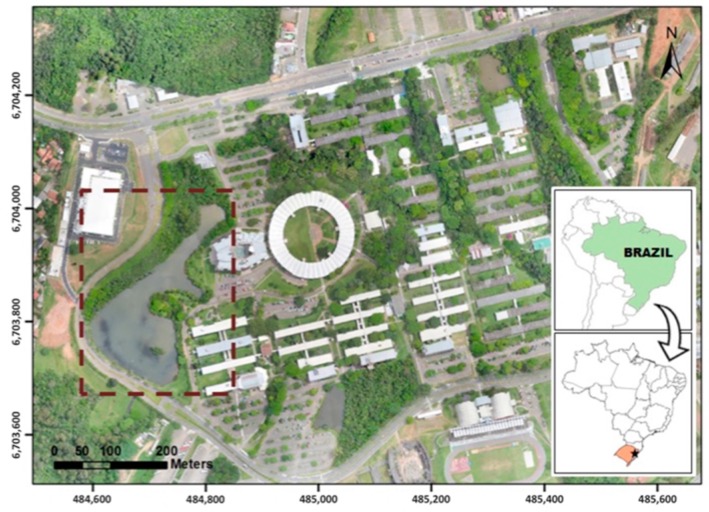
Study area.

The lake at Unisinos University, South Brazil, was selected as the study site (Figure 2). The lake is a small artificial water body and receives rainwater runoff of the campus, as it is located at the lowest altitude of the university’s terrain. Due to this, several inorganic and organic compounds reach this aquatic environment through the rainwater run-off into the drainage area. These compounds can be found in the lake in the form of suspended solids or dissolved organic matter. It is approximately 320 m and 170 m at its longest and widest points, respectively, with a surface area of approximately 25,000 m^2^ and a depth of 4 m at the center. This lake was chosen because the equipment and resources available at the university could be easily accessed for this study.

### 2.2. Data Acquisition

**Figure 3:**
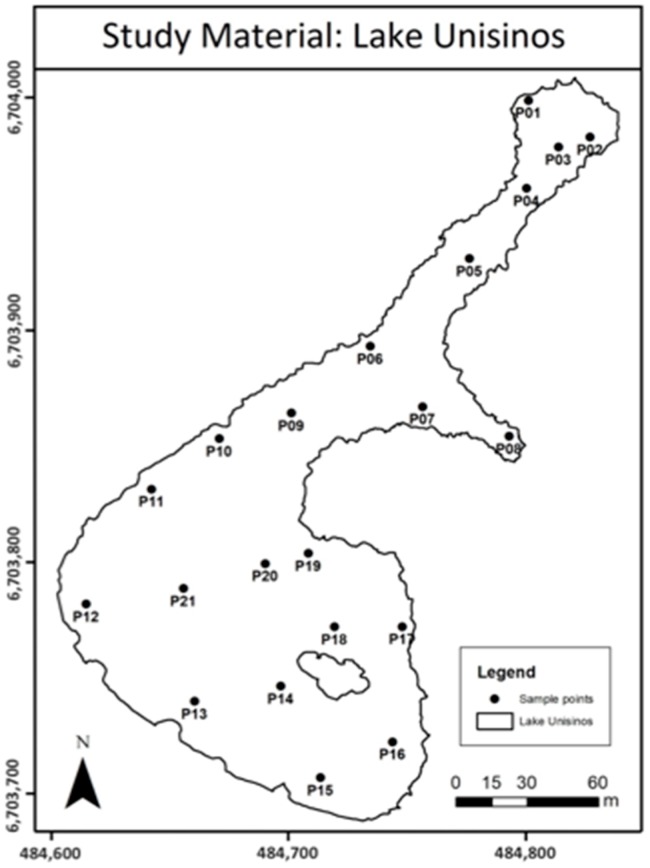
Positions of the sampling points.

Field sampling was performed in March 2017 and consisted of a UAV flight over the lake and an in situ collection of water samples at 21 sampling points, all occurring on the same day. Figure 3 shows the positions of the 21 sampling points.

The samplings were carried out in the superficial layer of water. The laboratory analysis for determination of total suspended solids (TSS) and dissolved organic matter (DOM) were made by the gravimetric method using the solids series (suspended and dissolved, fixed and volatile). DOM was obtained through the dissolved and volatile fraction of the samples. We sampled 5 L of water at each sampling point (a sample simple for each point), and the TSS and DOM analyses were performed in triplicate [26].

**Figure 4:**
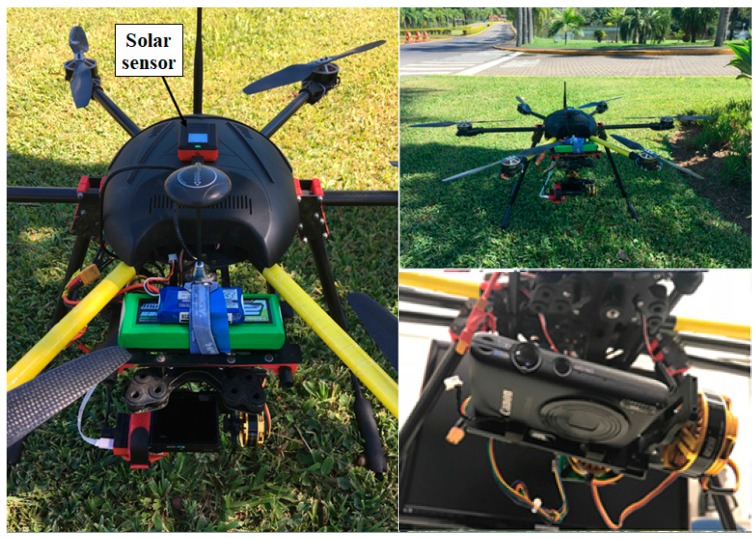
Hexacopter used for lake mapping.

The UAV used for image acquisition was a hexacopter (Figure 4), on which a Canon RGB sensor was mounted. Figure 4 presents the remote sensing system used in the acquisition of spectral images.

The UAV was flown at an altitude of 150 m with an average speed of 5 m/s, generating 46 images obtained with the Canon sensor. To georeference the images, we established a network with six ground control points (GCPs), evenly distributed throughout the study area, and obtained their geodetic coordinates by the real-time kinematic (RTK) method using the Global Navigation Satellite System (GNSS). We adopted SIRGAS 2000 (Geocentric Reference System for the Americas) as the referencing system in Universal Transverse Mercator (UTM) projection, zone 22S.

Processing of the UAV data was a simple, three-step workflow. In the first step, we created a controlled mosaic and determined the accuracy of the model after applying GCPs. In the second step, we created a point cloud from the pixels of the images using the structure from motion technique [27]. In the third step, we transformed the point cloud into a digital surface model (DSM) used as the basis for the orthorectification process. Thus, in terms of image quality, we generated an orthophoto with a pixel size of 0.04 × 0.04 m^2^.

### 2.3. Proposal of an Artificial Neural Network to Predict Spectral Bands of Images Acquired by a UAV

Neural networks were used in the following two modeling stages: ANN training and validation. Our working hypothesis is that it is possible to structure an ANN capable of predicting the spectral bands used in water quality monitoring in dams/lakes using the three bands of the Canon sensor (blue (B), green (G), and red (R)) as the ANN input. In order to structure the output of the network we used the bands *b*4 (0.64–0.67 μm), *b*5 (0.85–0.88 μm), and *b*9 (1.36–1.38 μm) of the LANDSAT 8 (OLI) satellite. The images were acquired from the United States National Aeronautics and Space Administration. We performed atmospheric correction of the images using the method described in [28,29]. We chose to predict bands *b*4, *b*5, and *b*9 because they are the most appropriate spectral bands to study water quality in dams and lakes [13,19].

As the images were georeferenced with the GNSS system, we standardized the pixel sizes of the images from the Canon sensor (0.04 m) based on those of the three bands of the satellite LANDSAT 8 (OLI) (30 m). Each pixel of the ANN input image corresponded to that of the output image. Thus, the B, G, and R Canon sensor images were the ANN input, and the *b*4, *b*5, and *b*9 LANDSAT 8 (OLI) images were the output. For ANN training, 80% of the pixels were used and, for validation, 20% were used, with randomly-defined data [30].

The ANN was trained using Adaptive Moment Estimation (Adam), which is an optimization algorithm that can be used instead of the classical stochastic gradient descent procedure to update network weights iteratively. Adam is simple to implement, computationally efficient, requires few memory requirements, has a good convergence rate, and is appropriate for machine learning problems [31,32].

During this training phase, as in most studies with neural networks [32], the definition of the best ANN topology was achieved through numerous experiments, in which the numbers of intermediate layers and neurons per layer, function of activation, and number of training cycles were tested. The activation function is important because it controls the way each individual neuron is activated and propagates the information through to the next layer. We used the sigmoid activation function because it increases the effectiveness of the backpropagation algorithm; thus, it is suitable for use in multilayer networks [33]. To ensure that the ANN achieved its results with the lowest possible amount of training effort, we normalized the intensity of the pixels to 0–1 [34].

After determining the topology that provided the lowest mean square error during supervised training, we validated the model with 20% of the data so that we can compare the known levels of intensity of the LANDSAT 8 (OLI) bands with those provided by the ANN.

### 2.4. Data Analysis

To evaluate the consistency of the results, we studied the correlation between the intensities of the known pixels from the ANN for each LANDSAT 8 (OLI) band. We calculated the coefficient of determination (R²) at a significance level of 5%.

Once the neural network was validated, we re-introduced the three-band Canon sensor images with their original resolution of 0.04 m^2^. By introducing the Canon sensor’s images into the ANN with their original resolution, we obtained the *b*4, *b*5, and *b*9 LANDSAT 8 (OLI) 8 images with pixels of 0.04 m^2^ as outputs. We georeferenced these images and generated the *NDVI* (Normalized Difference Vegetation Index) (Equation (1)) and *NDWI* (Normalized Difference Water Index) (Equation (2)):

(1) $$NDVI=\;\frac{b5-b4}{b5+b4}$$

(2) $$NDWI=\;\frac{G-b5}{G+b5}$$

*NDVI* was initially developed to evaluate vegetation biomass with higher reflectance in the near-infrared spectral range rather than in the red band. Thus, *NDVI* values vary between −1 and +1, with negative values corresponding to the presence of water, values close to zero representing exposed soil, and positive values indicating vegetated surfaces (ranging from not dense to dense, represented by an increase in the value of the index). *NDWI* was developed to measure the spectral characteristics of water using the green band in place of the red band, allowing water to be identified in terrestrial and vegetated surfaces. The *NDWI* values also vary from −1 to +1, with positive values indicating water bodies and negative values indicating vegetation and soil [35].

We plotted the sample points at which we obtained the total suspended solids (TSS) and dissolved organic matter (DOM) concentrations on the georeferenced *NDVI* and *NDWI* images. Four sample points were considered outliers because they were outside the range of two standard deviations (above or below) of the mean of the set [19]. They were excluded from the set, and the remaining seventeen data points for the analysis of correlations. We determined the correlations between TSS and DOM and the *NDVI* and *NDWI* values obtained from the images generated by the ANN.

## 3. Results and Discussion

In this section, we present the results of this research in three sub-sections: the results of laboratory analysis, the results of the ANN processing, and the correlations between TSS and DOM and the *NDVI* and *NDWI* values obtained from the images generated by the ANN.

### 3.1. Results of Laboratory Analysis

The laboratory analysis generated satisfactory results to determine the concentrations of TSS and DOM in water samples. Table 1 summarizes these results.

### 3.2. Results of Artificial Neural Network Processing

During the training process, using 80% of the pixels of LANDSAT (OLI) 8 and Canon sensor images, we tested different network structures, and varying numbers of intermediate layers, neurons per layer, and training cycles. Such a practical experimentation is common in studies that use neural networks [32,34] and the best topology identified was 3-4-6-3, with 100 training cycles at a computational cost of 288 s, considering the computer used had the following configuration: processor—Intel® Core ™ i7-3770 CPU @ 3.40 GHz ×8; memory—12 GB DDR3 1333 MHz RAM.

Once the best topology for the ANN was defined, we validated the ANN using 20% of the data from the pixels of LANDSAT (OLI) 8 and the Canon sensor images and compared the intensity of the known pixels (bands *b*4, *b*5, and *b*9) with those generated by the ANN. We adopted 80% of the data for training and 20% for validation, according to [30]. We calculated the mean square error and conducted a linear regression between the known pixel intensity values and those predicted by the ANN for the three output bands of the network. It is important to emphasize that we also evaluated nonlinear regression models. However, when evaluating the result of the adjustment by the least square method, the linear model was more effective than nonlinear models, presenting a higher value for the determination coefficient R^2^ and a lower value for the mean square error (MSR). The results are presented in Table 2.

**Figure 5:**
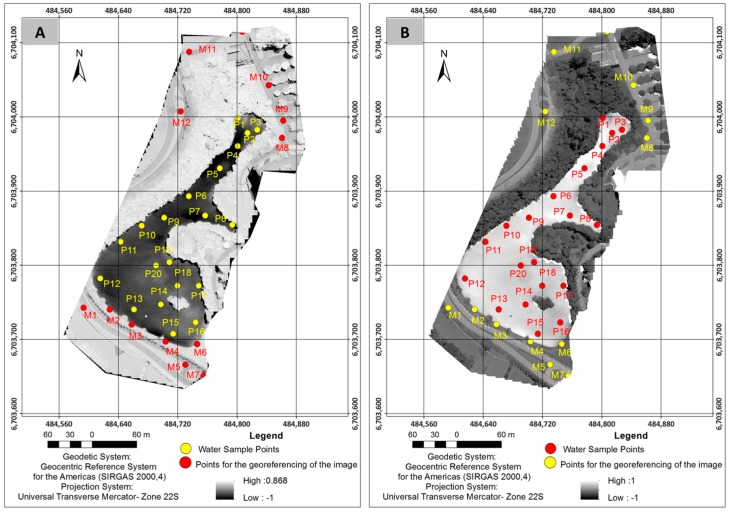
Images generated by the compositions of the spectral bands obtained by ANN: (**A**) *NDVI* image; and (**B**) *NDWI* image.

We used the Canon sensor images with their original resolutions (pixel of 0.04 m^2^) and introduced the three bands (B, G, R) in the trained and validated ANN, obtaining the three bands, *b*4, *b*5, and *b*9, with a computational cost of 30.5 s that generated *NDVI* and *NDWI* images (Figure 5).

### 3.3. Correlations

The results of the correlation analysis for *NDVI* and *NDWI* with the concentrations of TSS and DOM are presented in Table 3. The best results for the coefficient of determination (R^2^), root mean square error (RMSE), and standard deviation (SD) were from the polynomial regressions.

The results obtained and presented in Table 3 are consistent with those reported in previous studies. In the literature, R^2^ values of the correlations between *NDVI*/*NDWI* and TSS were around 0.60 [1,21]. In our study, R² values were 0.63 (*NDVI*) and 0.77 (*NDWI*), respectively. R^2^ values are higher in our study because our method allows the prediction of spectral bands with the same geometric resolution (pixel with centimetric size) as the resolution of bands R, G, and B used in the ANN input. For the correlations of *NDVI*/*NDWI* with DOM, our results are promising, but we did not find any studies with which to compare our results.

**Figure 6:**
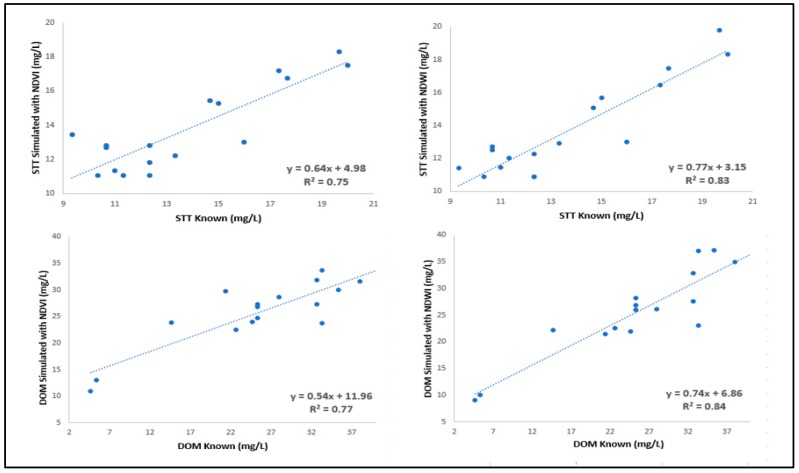
**S**catter plot of SST and DOM known and simulated considering the *b*4 and *b*5 bands in obtaining the *NDVI* and *NDWI* indices.

The consistency of the values shown in the Table 3 is evidenced when the scatter plot between the known and simulated values of TSS and DOM is established (Figure 6).

*NDVI* and *NDWI* are commonly used for images with near-infrared bands of approximately 800 to 900 nm [36]. Previous studies [37] mapped the spectral behavior of turbid water and water rich in suspended solids, and the spectral signature for TSS had reflectance peaks close to 550, 650, and 700 nm. The same patterns were found by other researchers [19]. However, water bodies in those studies have OACs with different characteristics, which make it possible to evaluate other compositions of spectral bands to determine *NDVI* and *NDWI*. For example, [38] proposed a method to obtain *NDWI* spectral bands ranging from 0.86 μm to 1.24 μm. In our lake, the TSS and DOM average concentrations are smaller than those in previous studies. Based on the work of [38] we modified Equations (1) and (2) and determined *NDVI* and *NDWI* using Equations (3) and (4):

(3) $$NDVI=\;\frac{b9-b4}{b9+b4}$$

(4) $$NDWI=\;\frac{G-b9}{G+b9}$$

**Figure 7:**
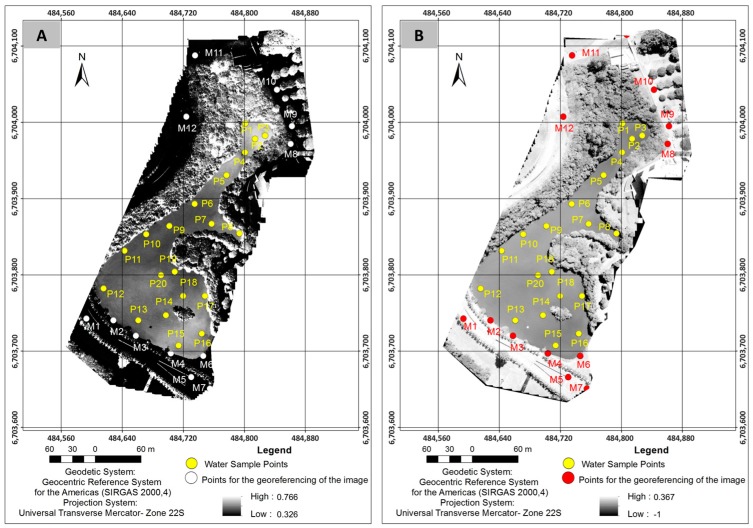
Images generated by the compositions of the spectral band *b*9 instead of the band *b*5 obtained by ANN: (**A**) *NDVI* image; and (**B**) *NDWI* image.

In Equations (3) and (4), we replaced band *b*5 (0.85–0.88 μm) with band *b*9 (1.36–1.38 μm) and extended the spectral range of the infrared to show the presence of TSS and DOM because mean concentrations of these elements were small (Figure 7).

Based on the *NDVI* and *NDWI* images shown in Figure 7, the results of correlation of these indices with TSS and DOM are presented in Table 4.

**Figure 8:**
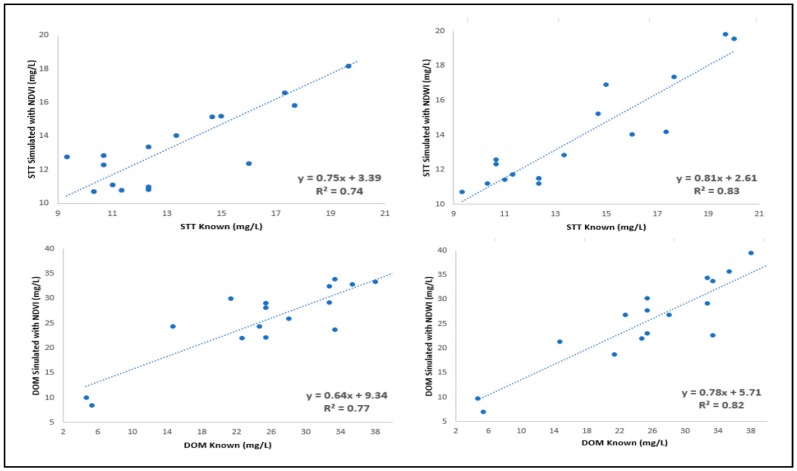
Scatter plot of SST and DOM known and simulated considering the *b*4 and *b*9 bands in obtaining the *NDVI* and *NDWI* indices.

Replacing *b*5 by *b*9 in *NDVI* and *NDWI* equations slightly increased the R² of the correlation of TSS and DOM with *NDVI*, and slightly decreased RMSE and SD (Table 4). The reason for the small increase is that band 9 provided a greater contrast of *NDVI*, indicating vegetative areas and the water body in Figure 7A. R^2^ of the correlation of TSS and DOM with *NDWI* showed a slight decrease. In Figure 7B, if we use the spectral band *b*9 instead of the *b*5 band, the contrast of the *NDWI* image worsened (compared to Figure 5B). The consistency of the results in Table 4 is also verified when the scatter plot between the known and simulated values of TSS and DOM is established (Figure 8).

**Figure 9:**
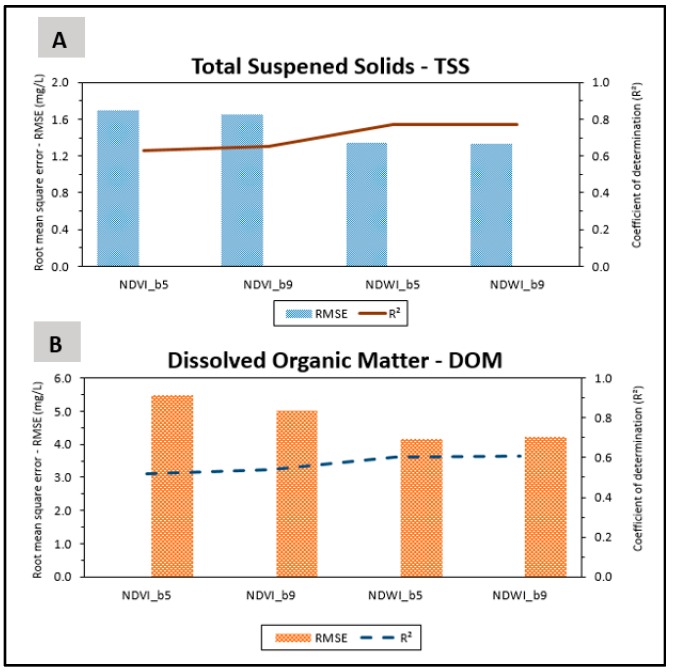
Comparison between the correlation results.

We synthesized all these results in Figure 9. The correlation of TSS with *NDVI* (Figure 9A) obtained with bands *b*5 and *b*9 show similar values for R^2^ (line), with values greater than 0.6, and for RMSE (bar chart), with values of approximately 1.7 mg/L. The correlation of TSS with *NDWI* showed a significant improvement in relation to the *NDVI*, with an increase of R² (around 0.77) and a decrease in RMSE (lower than 1.4 mg/L), also with similar results for bands *b*5 and *b*9. The results related to DOM (Figure 9B) followed the same trend as those related to TSS. We observed stronger correlations with *NDWI* (R^2^ of 0.60) and a significant decrease in RMSE compared to the correlations with *NDVI*.

The improvement in the results of the correlation with *NDWI* is in agreement with the results of Mohanty et al. [39] that used Indian remote sensing satellite images (IRS-IB) and the obtained R^2^ values between TSS and *NDWI* were around 0.6. Our results were higher than those of [39] because our method allows generating multispectral images (centimeter pixel size) from an expert system based on an ANN. The pixel size of multispectral images predicted by the ANN will always be equal to the pixel size of the images from a low-cost sensor (spectral bands R, G, B) because these are the bands used as ANN input data. The coefficients of correlation of TSS with *NDVI* in our study are also higher than those in Masocha et al. [40]. Our results for DOM were consistent, but we did not find any studies with which to compare our results.

Even with the good results found in this article, the proposed method has limitations that are highlighted below:

- The water sampling must be performed in periods under good light conditions. This condition is important to obtain high-quality UAV images and spectral bands of satellites without clouds;
- It can be difficult to obtain orthophotos by UAV for large dams/lakes. Generally, water bodies are quite homogeneous, making it difficult to determine the homologous points between the images during the processing. The homologous points are fundamental for creating a point cloud from the pixels of the images using the structure from motion technique and to transform the point cloud into a digital surface model (DSM) used as the basis for the orthorectification process [27];
- Our approach did not consider the seasonal variation of TSS and DOM. The homogeneity of the physical and chemical conditions of the water is a reflection of the temporal conditions of the bodies of water. Thus, our approach can be improved by incorporating in the neural network input the climate variable characteristics of the study site (for example, wind velocity, rainfall, temperature, etc.).

## 4. Conclusions

The results of this study demonstrate the applicability of a methodological model that uses an expert ANN system to monitor water quality by UAVs. We corroborate our hypothesis because we were able to predict the bands of a multispectral sensor through the insertion of bands from a low-cost sensor using an ANN validation process.

The results indicate that our proposed method for predicting multispectral bands based on an expert system and a low-cost sensor mounted on a UAV may be a useful tool for monitoring water quality for sites with low concentrations of TSS and DOM. Traditional methods of water quality monitoring in dams/lakes are time-consuming, costly, and often impractical in large bodies of water. Thus, the present study provides a better alternative for monitoring water quality using multispectral images.

For the water body we studied, the best spectral range to monitor TSS and DOM concentrations was 530–1380 nm. We established the correlations of TSS and DOM with *NDVI* and *NDWI* obtained from the spectral bands predicted by the ANN (*b*4: 0.64–0.67 μm), (*b*5: 0.85–0.88 μm), and (*b*9: 1.36–1.38 μm). The correlation coefficient for TSS was higher than that found for DOM and the R^2^ values were the highest for the correlations with *NDWI*. For correlations between TSS and the indices, R^2^ in this study was higher than that reported in the literature. Regarding the correlations with DOM concentrations, our results were consistent, but we could not find any studies to make comparisons.

The method described in this article can be applied to monitor other water bodies. With this method, it is easy to mount low-cost sensors on any unmanned aerial vehicle, making it possible to monitor water quality in dams and lakes.

## Figures and Tables

**Table 1:** Results of TSS and DOM analysis.

Parameter	Average (mg/L)	Minimum/Location (mg/L)	Maximum/Location (mg/L)	Standard Deviation (mg/L)
TSS	13.65	9.33 (P02)	20 (P15)	3.07
DOM	38.05	4.67 (P19)	175 (P12)	39.76

**Table 2:** Linear regression between known LANDSAT 8 (OLI) pixel intensity and the prediction from the proposed ANN model.

Pixel Intensity	Linear Equation ^(1)^	R^2^ ^(2)^	MSR ^(3)^
*b*4	y = 0.6271x + 31.2125	0.8210	6.80%
*b*5	y = 0.8051x + 20.0781	0.8401	18.15%
*b*9	y = 0.7097x + 25.4521	0.8120	10.37%

^(1)^ Linear regression conducted between known pixel intensity and ANN-predicted pixel intensity. ^(2)^ Determination coefficient. ^(3)^ Mean square error calculated by dividing the sum of the squared differences between known pixel intensities and ANN-predicted values per mean pixel intensity value.

**Table 3:** Polynomial regression results.

Correlation	Polynomial Equation	R^2^ ^(1)^	RMSE ^(2)^	SD ^(2)^
TSS × *NDVI*	TSS = 26.7 × *NDVI*^2^ + 30.9 × *NDVI* + 20.0	0.63	1.69	2.50
TSS × *NDWI*	TSS = 82.2 × *NDWI*^2^ − 131.5 × *NDWI* + 63.5	0.77	1.34	2.36
DOM × *NDVI*	DOM = 208.573 × *NDVI*^3^ + 229.6 × *NDVI*^2^ + 51.3 × *NDVI* + 27.0	0.52	5.48	3.87
DOM × *NDWI*	DOM = −1929.1 × *NDWI*^3^ + 3940.8 × *NDWI*² − 2592.4 × *NDWI* + 576.7	0.60	7.35	5.32

^(1)^ Correlations between the water quality parameters (TSS and DOM) and *NDVI* and *NDVI* indices obtained with the spectral bands *b*4 and *b*5 predicted by ANN. ^(2)^ Root mean square error (RMSE) and standard deviation (SD) are expressed as mg/L.

**Table 4:** Polynomial regression results.

Correlation	Polynomial Equation	R^2^ ^(1)^	RMSE ^(2)^	SD ^(2)^
TSS × *NDVI*	TSS = 45.4 × *NDVI*^2^ + 43.1 × *NDVI* + 20.9	0.65	1.65	2.33
TSS × *NDWI*	TSS = 68.7 × *NDWI*^2^ − 111.2 × *NDWI* + 56.1	0.76	1.33	2.54
DOM × *NDVI*	DOM = 244.9 × *NDVI*^3^ + 186.2 × *NDVI*^2^ + 7.0 × *NDVI* + 21.8	0.54	5.03	4.47
DOM × *NDWI*	DOM = −2119.5 × *NDWI*^3^ + 4559.1 × *NDWI*^2^ − 2760.4 × *NDWI* + 603.6	0.59	4.23	5.28

^(1)^ Correlations between the water quality parameters (TSS and DOM) and *NDVI* and *NDVI* indices obtained with the spectral bands *b*4 and *b*9 predicted by ANN. ^(2)^ Root mean square error (RMSE) and standard deviation (SD) are expressed as mg/L.
